# *Candida auris* in Heart Transplant Donor: First Isolation in a Southern Italy Heart Transplant Center

**DOI:** 10.1002/ccr3.71142

**Published:** 2026-01-17

**Authors:** Lorenzo Giovannico, Giuseppe Fischetti, Luca Savino, Giuseppina Caggiano, Maria Chironna, Silvio Tafuri, Tomaso Bottio

**Affiliations:** ^1^ Cardiac Surgery Unit University of Bari Aldo Moro Bari Italy; ^2^ Hygiene Section, Interdisciplinary Department of Medicine University of Bari Aldo Moro Bari Italy; ^3^ Microbiology and Virology Section, Interdisciplinary Department of Medicine University of Bari Aldo Moro Bari Italy

**Keywords:** **a**ntifungal prophylaxis, *Candida auris*, **d**onor screening, heart transplantation, **i**nfection control, **m**ultidrug resistance

## Abstract

*Candida auris* is an emerging multidrug‐resistant fungal pathogen increasingly implicated in healthcare‐associated outbreaks worldwide. Its presence in organ donors poses a significant threat to transplant recipients due to the risk of invasive infection and limited antifungal treatment options. We report the first isolation of *Candida auris* in a heart transplant donor at a transplant center in Southern Italy. The donor, a 45‐year‐old woman from Greece, was colonized with *C. auris* in the bronchoaspirate sample collected at the time of organ retrieval. Despite this colonization, the donor heart was successfully transplanted into a 62‐year‐old male recipient with end‐stage heart failure secondary to myocardial infarction and cardiogenic shock. The recipient received targeted perioperative prophylaxis and was placed under strict isolation protocols. Repeated microbiological surveillance, including blood, urine, and mucosal cultures, revealed no evidence of *C. auris* transmission. Environmental surveillance of the operating room and ICU also tested negative. The patient recovered uneventfully, showing good cardiac function and no signs of graft rejection or infection. This case emphasizes the critical importance of early detection, thorough microbiological assessment, and stringent infection control in transplantation involving donors colonized with multidrug‐resistant organisms. It also raises the question of whether *C. auris* should be routinely screened in potential donors and if specific transplant guidelines should be developed to address such emerging threats.

## Graphical abstract

Timeline of infection control in a heart transplant from a donor colonized with Candida auris: from detection at retrieval to strict isolation, antifungal prophylaxis, negative follow‐up cultures, and successful prevention of transmission.

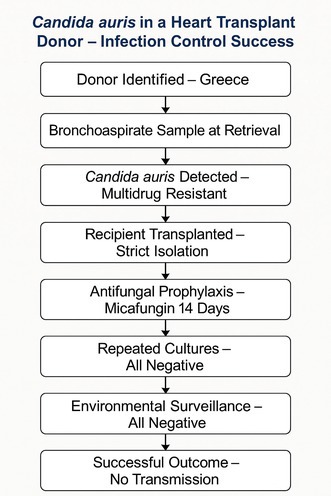

**Summary**

- *Candida auris*, a multidrug‐resistant fungal pathogen, was identified in a heart donor in Southern Italy.
- Rigorous infection control and microbiological surveillance successfully prevented transmission.
- This case highlights the need for systematic donor screening and infection prevention strategies in transplant programs facing emerging fungal threats.

## 1 Introduction

Heart failure (HF) is a global health issue, affecting over 64.3 million people worldwide in 2017. Its prevalence is expected to rise due to improved survival following HF diagnosis and increasing life expectancy [1, 2]. Heart transplantation remains the gold standard for end‐stage HF patients, despite the limited availability of donors. The use of marginal donors, when carefully matched with recipients, is a viable option [3, 4].

This report presents the first case of *Candida auris* isolation in a heart transplant donor in southern Italy, emphasizing the importance of microbiological surveillance and infection control measures in transplant settings.

## 2 Case History/Examination

A 62‐year‐old man with a history of arterial hypertension and smoking habits presented to the emergency room with epigastric pain, asthenia, and hypotension. He was diagnosed with inferior myocardial infarction and underwent emergency angioplasty with stenting.

Despite the intervention, his cardiac function did not improve, and he experienced frequent ventricular tachycardia episodes. Three days postinfarction, he developed ventricular fibrillation, which was resuscitated after cardiac defibrillation. Due to cardiogenic shock, he was transferred to the cardiac surgery unit for veno‐arterial extracorporeal membrane oxygenation (V‐A ECMO) and emergency heart transplant listing.

## 3 Differential Diagnosis, Investigations, and Treatment

A donor was identified 4 days later: a 45‐year‐old woman from Greece with matching anthropometric parameters. Her blood tests showed normal myocardial necrosis indices, an echocardiogram revealed preserved function (EF 60%, TAPSE 22 mm), and coronary angiography was normal.

Although the donor remained afebrile, leukocytosis and increased inflammation indices were noted. Blood cultures were negative, but bronchocultures were positive for *Acinetobacter baumannii* and *Klebsiella pneumoniae* . Based on these findings, the national transplant center approved the heart for transplantation but required additional microbiological investigations at the time of organ retrieval. Lungs and other organs were not allocated due to colonization risk and microbiological findings. All centers receiving organs from this donor were promptly informed about the detection of *Candida auris* to ensure appropriate posttransplant monitoring and infection control protocols.

The recipient underwent orthotopic heart transplant using the standard bicaval technique (ischemic time: 4 h). Postoperatively, he received prophylactic antibiotics (Piperacillin/Tazobactam and Vancomycin) and was initiated on an immunosuppression protocol.

## 4 Conclusion and Results (Outcome and Follow‐Up)

The microbiology laboratory later reported that the donor's blood and urine cultures were negative, but the bronchoaspirate was positive for *Candida auris*. Antifungal susceptibility testing showed high resistance to fluconazole (> 256 g/mL) and variable susceptibility to other antifungal agents. Identification of *Candida auris* was performed using matrix‐assisted laser desorption ionization–time of flight mass spectrometry (MALDI‐TOF MS; Bruker Daltonics). Colonies were grown on CHROMagar Candida medium and incubated at 37°C for 48 h. Identification was confirmed by the species‐specific spectral profile in the Bruker Biotyper database (DB‐5989). Although MALDI‐TOF MS was used in our laboratory, we acknowledge that sequencing‐based methods such as ITS region amplification remain the gold standard for species‐level identification of rare yeasts.

Given the isolation of *Candida auris* in the donor's bronchoaspirate, the recipient was started on antifungal prophylaxis with micafungin (100 mg daily intravenously), initiated immediately after transplantation and continued for 14 days. (Table 1) This choice was based on the antifungal susceptibility profile and current recommendations for echinocandin use in *C. auris* colonization. No adverse events or breakthrough fungal infections occurred during or after prophylaxis.

**TABLE 1:** Antifungal susceptibility profile of *Candida auris* isolate.

Antifungal agent	MIC (μg/mL)	Interpretation
Fluconazole	> 256	Resistant
Voriconazole	2	Intermediate
Amphotericin B	1	Intermediate/Resistant^a^
Caspofungin	0.25	Susceptible
Micafungin	0.12	Susceptible
Anidulafungin	0.25	Susceptible

^a^ Interpretive breakpoints for *Candida auris* are based on CDC tentative MIC thresholds due to limited clinical data.

Despite donor colonization, strict infection control measures prevented transmission to the recipient. He was placed in isolation and monitored for 15 days with repeated cultures (urine, axillary swabs, wound, and mucocutaneous swabs), all of which remained negative.

Environmental surveillance was conducted in the operating room and ICU, with swabs taken from medical devices and high‐touch surfaces, all testing negative for *Candida auris*.

The patient was later transferred to a regular ward, achieving good functional recovery. Echocardiography confirmed preserved biventricular function and the absence of valve disease. After four endomyocardial biopsies ruling out rejection (ISHLT'04 0R), he was discharged in excellent condition.

## 5 Discussion

*Candida auris*, a yeast species first isolated in 2009 in Japan, is known for its multidrug resistance and potential to cause invasive infections, including bloodstream infections with high mortality rates (30%–60%). Most strains exhibit resistance to at least one major antifungal class, with some strains resistant to all three major classes (azoles, echinocandins, and polyenes) [5, 6, 7].

The first Italian case was reported in July 2019, with an outbreak in northern regions leading to 361 cases by December 2022. Our case represents the first documented *Candida auris* isolation in a heart transplant donor in southern Italy.

*Candida auris* can persist on surfaces and medical devices for extended periods, complicating infection control. Consequently, following Italian Ministry of Health recommendations, we performed rigorous environmental surveillance, which confirmed the absence of contamination. Genotyping of the *Candida auris* isolate, such as clade identification via whole‐genome sequencing or ITS sequencing, was not performed due to logistical constraints at our center [8]. However, given the donor's origin from Greece—a country with documented cases of *C. auris* —we hypothesize that the strain may belong to the South Asian or African clade, both previously reported in southern Europe. Future cases should incorporate molecular typing to track potential transmission routes and clade‐specific resistance patterns.

This case highlights the importance of strict infection control measures in transplant centers, especially when dealing with multidrug‐resistant organisms. Despite the donor's colonization, proper organ procurement and handling procedures prevented transmission, reinforcing the effectiveness of existing protocols.

## 6 Conclusion

This case demonstrates that successful heart transplantation from a *Candida auris*‐colonized donor is possible when rigorous infection prevention and microbiological monitoring protocols are implemented. The absence of transmission to the recipient underscores the effectiveness of strict isolation measures, targeted prophylaxis, and postoperative surveillance.

Given the global emergence of *C. auris* and its multidrug resistance, this case supports the need to include *C. auris* in routine microbiological assessments of organ donors, particularly those with risk factors or originating from high‐prevalence regions.

Moreover, early detection, thorough environmental decontamination, and clinical vigilance are essential to minimizing transmission risk and ensuring patient safety. Transplant centers should consider developing specific guidelines for managing colonized donors to standardize care in the context of emerging infectious threats.

## Author Contributions

**Lorenzo Giovannico:** conceptualization, writing – original draft, writing – review and editing. **Giuseppe Fischetti:** writing – original draft. **Luca Savino:** writing – original draft. **Giuseppina Caggiano:** supervision, writing – review and editing. **Maria Chironna:** supervision, writing – review and editing. **Silvio Tafuri:** supervision, writing – review and editing. **Tomaso Bottio:** formal analysis, supervision, writing – review and editing.

## Consent

The authors confirm that written informed consent has been obtained from the involved patient.

## Conflicts of Interest

The authors declare no conflicts of interest.

## Data Availability

The data that support the findings of this study are available from the corresponding author, L.G., upon reasonable request.

## References

[1] T. A. McDonagh , M. Metra , M. Adamo , et al., “2021 ESC Guidelines for the Diagnosis and Treatment of Acute and Chronic Heart Failure,” European Heart Journal 42, no. 36 (2021): 3599–3726. 34447992 10.1093/eurheartj/ehab368

[2] G. Savarese , P. M. Becher , L. H. Lund , P. Seferovic , G. M. Rosano , and A. J. Coats , “Global Burden of Heart Failure: A Comprehensive Review,” Cardiovascular Research 118, no. 17 (2023): 3272–3287. 35150240 10.1093/cvr/cvac013

[3] O. Bifulco , T. Bottio , R. Caraffa , et al., “Marginal Versus Standard Donors in Heart Transplantation,” Journal of Clinical Medicine 11 (2022): 2665. 35566789 10.3390/jcm11092665 PMC9105473

[4] L. Giovannico , D. Parigino , A. D.'E. Ramirez , et al., “World's Oldest Heart Transplant Donor: Age Is Just a Number,” Journal of Cardiovascular Medicine 25, no. 3 (2024): 243–245. 38305142 10.2459/JCM.0000000000001585

[5] K. Satoh , K. Makimura , Y. Hasumi , Y. Nishiyama , K. Uchida , and H. Yamaguchi , “Candida auris sp. Nov,” Microbiology and Immunology 53, no. 1 (2009): 41–44. 19161556 10.1111/j.1348-0421.2008.00083.x

[6] C. Sticchi , R. Raso , L. Ferrara , et al., “Increasing Cases of *Candida auris* in North Italy,” Journal of Clinical Medicine 12, no. 5 (2023): 1912. 36902700 10.3390/jcm12051912 PMC10003924

[7] D. R. Giacobbe , L. Magnasco , C. Sepulcri , et al., “Advances in *Candida auris* Treatment,” Expert Review of Clinical Pharmacology 14, no. 10 (2021): 1205–1220. 34176393 10.1080/17512433.2021.1949285

[8] A. P. Magiorakos , K. Burns , J. Rodríguez Baño , et al., “Infection Prevention and Control Measures and Tools for the Prevention of Entry of Carbapenem‐Resistant Enterobacteriaceae into Healthcare Settings: Guidance from the European Centre for Disease Prevention and Control,” Antimicrobial Resistance and Infection Control 6 (2017): 113, 10.1186/s13756-017-0259-z. 29163939 PMC5686856

